# Developing a risk assessment tool for cancer-related venous thrombosis in China: a modified Delphi-analytic hierarchy process study

**DOI:** 10.1186/s12885-024-11877-8

**Published:** 2024-01-23

**Authors:** Xiaoli Qin, Xiurong Gao, Yujie Yang, Shunlong Ou, Jing Luo, Hua Wei, Qian Jiang

**Affiliations:** 1 Department of Pharmacy, The Third People’s Hospital of Chengdu, 610031 Chengdu, Sichuan P.R. China; 2 School of Pharmacy, Chengdu Medical College, 610500 Chengdu, Sichuan P.R. China; 3 Department of Pharmacy, Sichuan Clinical Research Center for Cancer, Sichuan Cancer Hospital & Institute, Sichuan Cancer Center, Affiliated Cancer Hospital of University of Electronic Science and Technology of China, 610041 Chengdu, Sichuan P.R. China; 4 Department of Pharmacy, The Second People’s Hospital of Yibin, 644000 Yibin, Sichuan P.R. China; 5 Department of Pharmacy, Chengdu Second People’s Hospital, 610011 Chengdu, Sichuan P.R. China

**Keywords:** Risk assessment tool, Cancer-related VTE, Venous thromboembolism, Delphi method, Analytic Hierarchy process

## Abstract

**Objective:**

To develop a Risk Assessment Tool for Cancer-related Venous Thrombosis in China.

**Methods:**

A modified two-round Delphi method was employed to establish consensus within a field to reach an agreement via a questionnaire or by interviewing a multidisciplinary panel of experts by collecting their feedback to inform the next round, exchanging their knowledge, experience, and opinions anonymously, and resolving uncertainties. Furthermore, The AHP (Analytic Hierarchy Process) was used to determine the final quality indicators’ relative importance.

**Results:**

The expert’s positive coefficient was 85.19% in the first round and 82.61% in the second round, with authoritative coefficients of 0.89 and 0.92 in the respective surveys. The *P*-value of *Kendall’s W* test was all less than 0.001 for each round, and the *W-value* for concordance at the end of the two rounds was 0.115. The final Risk Assessment Tool for Cancer-related Venous Thrombosis consisted of three domains, ten subdomains, and 39 indicators, with patient factors weighing 0.1976, disease factors weighing 0.4905, and therapeutic factors weighing 0.3119.

**Conclusion:**

The tool is significantly valid and reliable with a strong authority and coordination degree, and it can be used to assess the risk of cancer-related VTE and initiate appropriate thrombophylactic interventions in China.

## Introduction

Venous thromboembolism (VTE), a medical condition that encompasses pulmonary embolism (P.E.) and deep vein thrombosis (DVT), is the second most common condition after acute myocardial infarction and stroke [1]. It has become a significant cause of unexpected death and a common complication for cancer patients. Previous studies have demonstrated that individuals with Cancer have a higher risk of developing thrombosis, which was 4–7 times greater than those without Cancer and accounted for approximately 20% of all VTE cases [2, 3]. Nevertheless, the occurrence and mortality rate of thrombosis in patients can be lowered with proper evaluation and early prevention [4]. It is important to note that excessive prevention may lead to a higher risk of bleeding and financial burden. As a result, it is crucial to utilize appropriate risk assessment tools to forecast venous thromboembolism associated with Cancer.

There have been several cancer-related VTE assessment tools developed from 2008 to 2021, including 5 in the United States, 2 in Italy, 2 in Spain, and 1 in China [5–18]. One of the initial risk assessment tools for VTE in outpatients undergoing chemotherapy was developed by Alok A. Khorana et al. in 2008 [5]. However, previous evaluations [19] have shown that these existing tools are highly biased, and their verification results vary significantly. The overall methodological quality of these tools needs improvement, and there is a need to study their risk stratification ability. Furthermore, these tools’ lack of reliability and validity is crucial for patient outcomes such as morbidity and mortality. As mentioned, China’s clinical practice still has limitations, particularly regarding systematic and practical VTE risk assessment tools for cancer patients.

The Delphi method effectively gathered expert opinions by providing anonymity, feedback, and statistical analysis, which led to a consensus through repeated information exchange and feedback. However, this approach cannot compare the importance of these indicators. The Analytic Hierarchy Process (AHP) is a method that can be used to compare the importance of indicators through weight analysis. This method complements indicators by systematically assigning weights to each indicator based on relative importance. Decision-makers can make more informed and rational decisions by considering the weights assigned to each indicator, enhancing the overall quality and reliability of the decision-making process. This study combined a modified Delphi method and AHP to devise a cancer-related VTE risk assessment tool in China.

## Methods

### Literature retrieval and construction of preliminary index pool

**Fig. 1:**
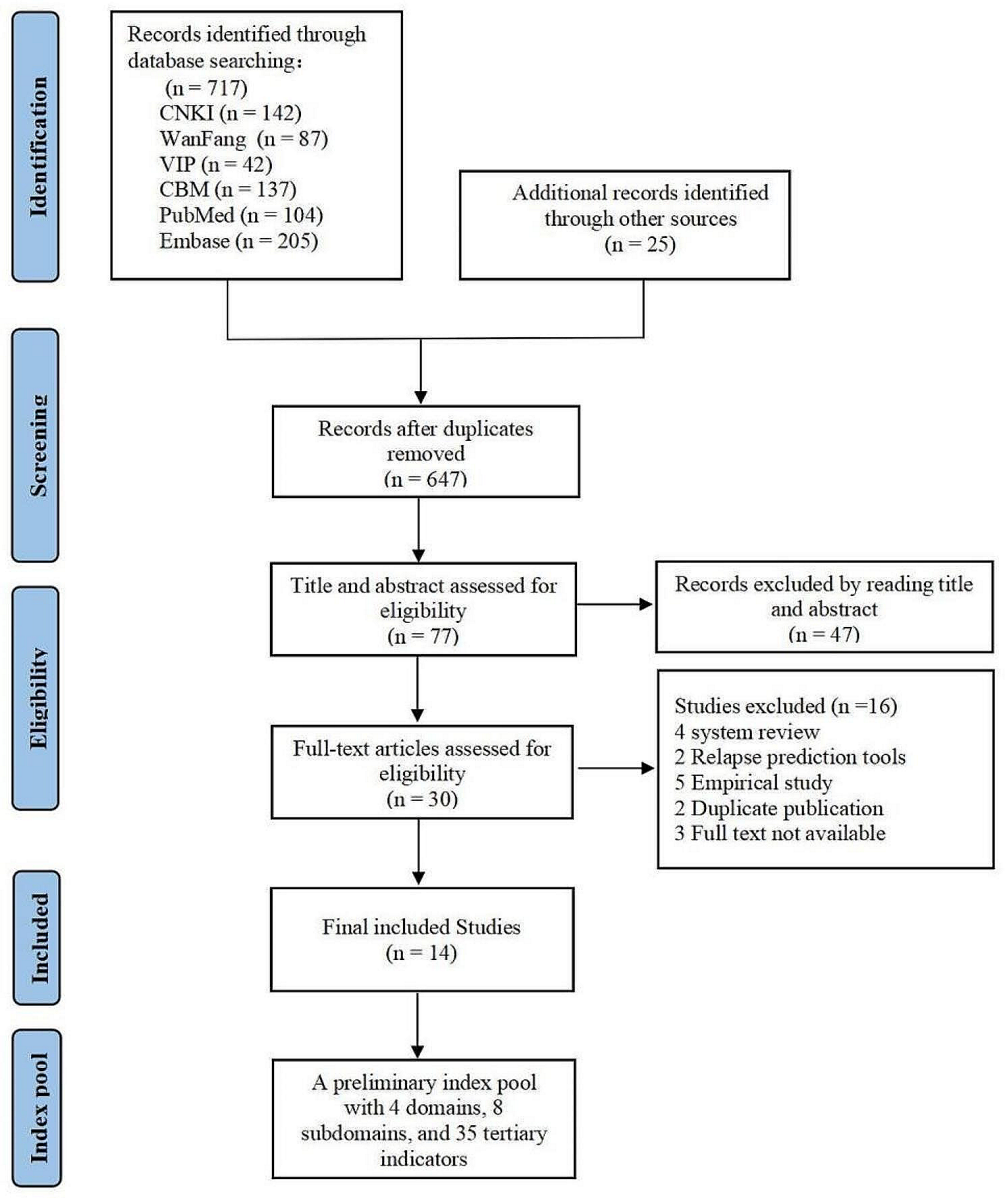
The flow chart of searching the literature

A literature retrieval was conducted to identify the risk factors for cancer-related VTE. The search encompassed several databases, including CNKI, WanFang, VIP, CBM, PubMed, Embase, and 21 related institutions’ and societies’ websites, and it continued until January 2023. Additionally, the references of relevant studies were manually searched to ensure that all critical information was included. English and Chinese search terms were utilized, including Venous Thromboembolism, Deep Vein Thrombosis, Pulmonary Embolism, cancer, tumor, neoplasms, risk assessment tool/score/model, risk evaluation tool/score/model. The 21 websites of related institutions and societies included 15 health-related websites in China, the United States, Japan, Canada, and other countries, as well as specific medical associations and societies such as the Chinese Medical Association, Chinese Society of Clinical Oncology(CSCO), National Comprehensive Cancer Network (NCCN), American Society of Clinical Oncology (ASCO), European Society for Medical Oncology (ESMO), and International Society of Thrombosis and Hemostasis (IGTH), for a total of 6 society websites. Based on the references and discussion in the group, the research referred to the existing and commonly used VTE risk assessment tools, combined with the actual situation in China, and developed a risk item pool. Finally, an initial preliminary index pool for cancer-related VTE was formed, including 4 first-level items, 8 second-level items, and 35 third-level items. The flow chart of searching the literature is shown in Fig. 1.

### Study design

**Fig. 2:**
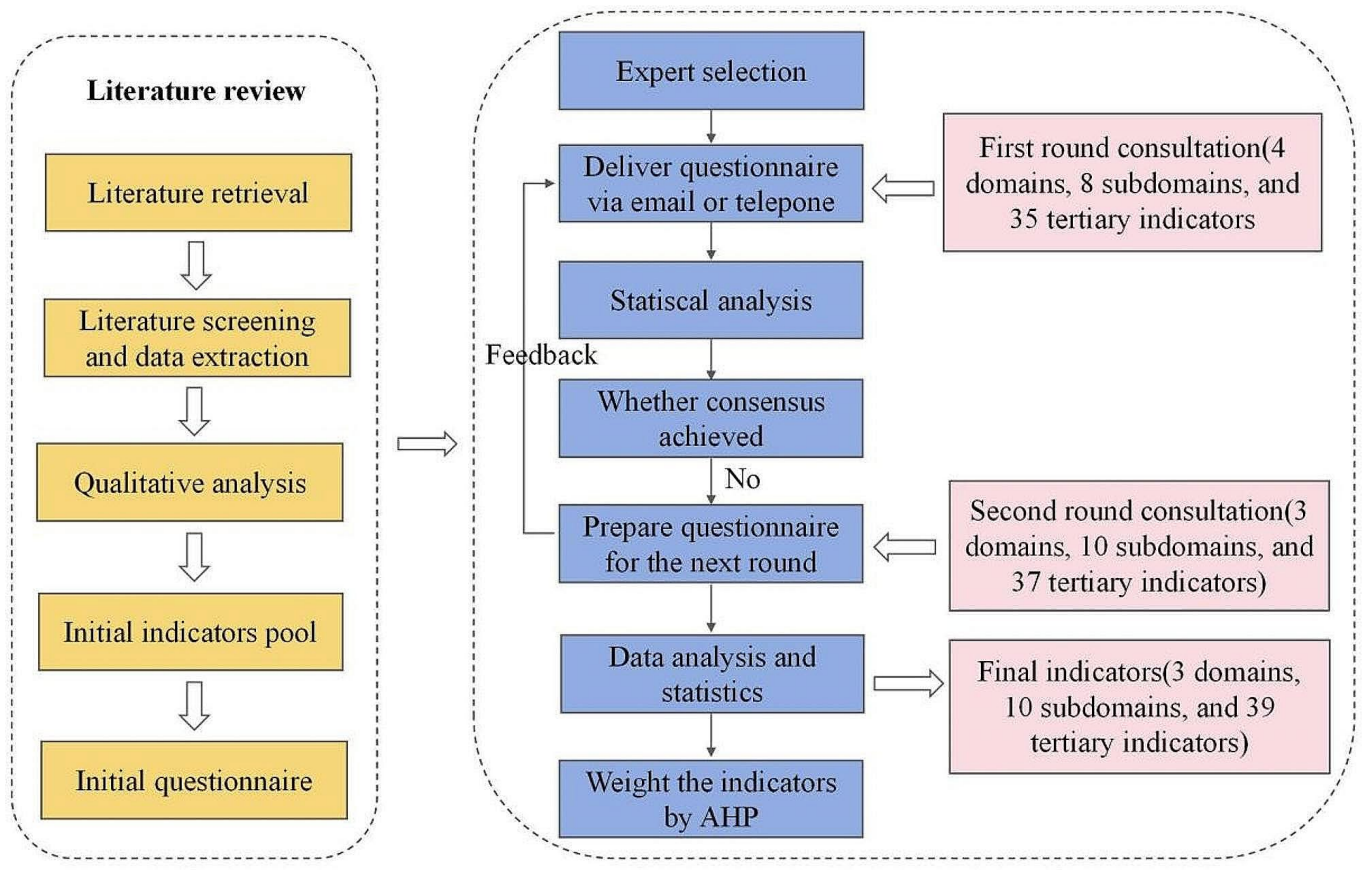
A study design

In the early stage, we obtained a preliminary index pool through a literature review and formed a preliminary correspondence form after discussion by the research group. After two rounds of Delphi expert consultation, the risk predictors of cancer-related VTE were obtained, and AHP analyzed the index weights. The detailed design process is shown in Fig. 2.

### Expert selection

A distinguished group of 27 professionals, comprising clinical, pharmaceutical, and nursing experts from Level A general hospitals, were chosen as Delphi consultation experts. These experts met stringent criteria, including over a decade of work experience in their field, a bachelor’s degree or above, the title of an associate professor or higher, familiarity with the research topic, and a willingness to participate in multiple rounds of Delphi consultation communication.

### Data collection

A group of researchers created indicators for cancer-related VTE risk using evidence-based research. This scale included four first-level items, eight second-level items, and 35 third-level items. Experts were contacted via e-mail or telephone and provided a questionnaire outlining the research background, objectives, and methodology. The questionnaire gathered information in four areas: *i*) demographic information of the experts, such as years of work, education, and research field. *ii*) Delphi expert consultation content, where each expert rated the necessity, importance, and operability of 47 potential indicators using a Likert scale from 1 to 5. *iii*)A familiarity scale ranging from 1 (unfamiliar) to 5 (very familiar), and *i*v) a basis for their judgments, which included theoretical analysis, experience, peer understanding, and personal intuition.

Throughout each round, experts were allowed to ask open questions and suggest changes to the index system by revising, deleting, or adding indicators. After a comprehensive analysis, We have made adjustments to the indicators accordingly. Additionally, the questionnaire was modified based on the previous round’s qualitative feedback and statistical analysis. After reaching a consensus, we established the final index system.

### Data analysis

The data was analyzed using the SPSS 22.0 statistical software and the YAAHP 10.1 analytic hierarchy process software. To be included in the analysis, items had to receive a necessity, importance, and operability score of ≥ 4.0 points, achieve a percentage of full score over 50%, and have a variation coefficient<0.25. The Analytic Hierarchy Process (AHP) was used to establish the hierarchical structure and construct the judgment matrix. The values were determined based on the average ratings of each criterion using Satya’s scale. This process helped in determining the criteria. At last, the combined weight of third-level items was multiplied by 100 and rounded to an integer value, representing the risk score of the corresponding three-level items for practical purposes.

## Results

### Characteristics of the experts

This study surveyed 23 experts from various regions of China, including Sichuan (18, 78.26%), Chongqing (2, 8.7%), Guangdong (1, 4.35%), Guizhou (1, 4.35%), Yunnan (1, 4.35%), and et al., with diverse occupations. Of the participants, 11 (47.83%) were clinicians, 10 (43.48%) were pharmacy staff, and 2 (8.7%) were nursing staff. The educational background of the specialists varied, with 14 (60.87%) holding a master’s degree, 6 (26.09%) holding a Ph.D, and 3 (13.04%) holding a bachelor’s degree. All professionals held senior or associate senior titles, with 9 (39.13%) seniors and 14 (60.87%) associate seniors. More information about the demographics of the experts is available in Table 1.

**Table 1:** Characteristics of expert panelists(*n* = 23)

Categories	Characteristics	Number	Percentage(%)
Response to questionnaires	Round1	23	85.19
	Round2	19	82.61
Gender	Male	11	47.83
	Female	12	52.17
Educational attainment	Doctor’s degree	6	26.09
	Master’s degree	14	60.87
	Bachelor’s degree	3	13.04
Types of expertise	clinicians	11	47.83
	pharmacists	10	43.48
	nurses	2	8.70
Main research areas	Clinical Pharmacy	4	17.39
	pharmaceutical affairs	6	26.09
	Oncology	3	13.04
	Internal Medicine	4	17.39
	surgery	1	4.35
	intensive care	1	4.35
	Critical Care Medicine	1	4.35
	neurology	1	4.35
	pediatrics	1	4.35
	hematology	1	4.35
Professional title	Senior	9	39.13
	Associate senior	14	60.87
Professional years	20~	7	30.43
	10~20	16	69.57
province or region	Sichuan	18	78.26
	Chongqing	2	8.70
	Guangdong	1	4.35
	Guizhou	1	4.35
	Yunnan	1	4.35
Familiarity degree	Very Familiar	5	21.74
	Familiar	15	65.22
	Familiar commonly	3	13.04

### Expert’s authority coefficient

The reliability of consulting outcomes is measured by the Expert’s Authority Coefficient (*Cr*), which is taken into account Expert Familiarity (*Cs*) and Judgment Basis (*Ca*) [20]. During the initial round, 87.0% (47 indicators) of the outcomes had a *Cr* of ≥ 0.80(ranging from 0.73 to 0.99, mean = 0.89). The second round had a slightly higher average *Cr* of 0.90 (ranging from 0.78 to 1.00) than the first. (Table 2)

**Table 2:** The values of *Cr* in two surveys

	Round one	Round two
*Cs*	0.86 ± 0.10	0.88 ± 0.09
*Ca*	0.92 ± 0.07	0.93 ± 0.08
*Cr*	0.89 ± 0.07	0.90 ± 0.07

### Degree of coordination of experts’ opinions

In the first round, the coefficient of variation (*CV*) for the 44 indicators’ necessity was<0.25 (ranging from 0.00 to 0.24, mean = 0.16). Meanwhile, the *CV* for the six indicators in importance was ≥ 0.25, and the *CV* for the 44 indicators’ operability was<0.25 (ranging from 0.00 to 0.23, mean = 0.14). The *Kendall’s W* coefficient was 0.185, 0.190 and 0.159. Finally, we would filter indicators by combining *CV*, mean, and full score ratio. Table 3 for details.

**Table 3:** Coordination degree of experts’ opinions in the two-round survey

Rounds			Round one			Round two		
Categories	*CV*	*W*-value	χ^2^	*P*-value	*CV*	*W*-value	χ^2^	*P*-value
necessity	0.16	0.185	196.04	0.000	0.12	0.134	111.533	<0.001
importance	0.16	0.190	201.489	0.000	0.13	0.132	116.812	<0.001
operability	0.14	0.159	168.669	<0.001	0.10	0.079	73.268	0.014

### Weight and score of risk factors for cancer-related VTE

We consulted with experts twice and reached a consensus on the final risk assessment for cancer-related VTE, comprised of 3 domains, ten subdomains, and 39 indicators. We used the average random consistency index *R.I.* to determine the matrix’s consistency at various levels. The results indicated that every matrix’s consistent ratio *(C.R.)* value *<* 0.1 meets the consistency test requirements. (Table 4)

**Table 4:** Weight Value and Score for the Indicators

Indicators	Weight value	Combined weight value	Maximum eigenvalue (max)	C.R.	Score
**Domains**			3.0537	0.0517	
1. Patient factors	0.1976				
2. Disease factors	0.4905				
3. Therapeutic factors	0.3119				
**Subdomains**					
1. Patient factors	0.1976		4.0610	0.0229	
1.1 basic feature	0.2002	0.0396			
1.2 complication	0.3290	0.0650			
1.3 Past medical history	0.1418	0.0280			
1.4 Inspection indicators	0.3290	0.0650			
2. Disease factors	0.4905		3.0000	0.0000	
2.1Tumor diagnosis time	0.2500	0.1226			
2.2Primary tumor site	0.5000	0.2452			
2.3Tumor staging	0.2500	0.1226			
3. Therapeutic factors	0.3119		3.0183	0.0176	
3.1medication	0.5571	0.1738			
3.2surgical treatment	0.3202	0.0999			
3.3Other treatments	0.1226	0.0382			
**Indicators**					
1.1.1Asian population	0.1153	0.0046			-1
1.1.2 BMI>24 kg/m2	0.1492	0.0059			1
1.1.3 BMI ≥ 28 kg/m2	0.2442	0.0097			2
1.1.4 Age ≥ 60 years old	0.3329	0.0132			1
1.1.5 female	0.0792	0.0031			1
1.1.6Permanent residence: Plateau (≥ 2500 m)	0.0792	0.0031			1
1.2.1 Decreased activity ability	0.3290	0.0214			2
1.2.2 cardiovascular disease	0.3290	0.0214			2
1.2.3 Lung or kidney disease	0.2002	0.013			1
1.2.4 Severe infection	0.1418	0.0092			1
1.3.1 VTE Personal History	0.4893	0.0137			1
1.3.2 VTE Family History	0.1381	0.0039			1
1.3.4 Hospitalization history (≤ 1 month)	0.1381	0.0039			1
1.3.5 History of lower limb varicose veins	0.2345	0.0066			1
1.4.1 D-Dimer ≥ 500ug/L	0.5000	0.0325			3
1.4.2 Plt ≥ 350 × 109/L	0.2500	0.0163			2
1.4.3 TAT>4ng/mL	0.2500	0.0163			2
2.1.1Cancer diagnosis time ≤ 6months	0.1226	0.0150			1
2.2.1 stomach	0.2500	0.0613			6
2.2.2 pancreas	0.2500	0.0613			6
2.2.3 lung	0.1250	0.0307			3
2.2.4 lymphoma	0.1250	0.0307			3
2.2.5 gynaecology	0.1250	0.0307			3
2.2.6 urogenital system	0.1250	0.0307			3
2.3.1 CancerCancer compresses blood vessels/lymphatic tissue	0.3917	0.048			5
2.3.2 transfer	0.2792	0.0342			3
2.3.3 Invasion of lymph nodes	0.1646	0.0202			2
2.3.4Mediastinal involvement	0.1646	0.0202			2
3.1.1 platinum	0.0537	0.0093			1
3.1.2Hormonal replacement therapy	0.1594	0.0277			3
3.1.3 High dose dexamethasone (≥ 160 mg/cycle)	0.0981	0.017			2
3.1.4 Antiangiogenic drugs	0.1035	0.018			2
3.1.5 Anticoagulants	0.3233	0.0562			-6
3.1.6 Adriamycin	0.0790	0.0137			1
3.1.7 Erythropoietic stimulating factor	0.1832	0.0318			3
3.2.1 Surgical site (hip or knee surgery)	0.4905	0.049			5
3.2.2 Surgical time ≥ 2 h	0.3119	0.0312			3
3.2.3 Surgical type (laparoscopic surgery, arthroscopic surgery)	0.1976	0.0197			2
3.3.1 CVC	0.0382	0.0015			1

Note: TAT: Thrombin antithrombin complex; CVC: Central venous catheterization

## Discussion

In this study, we developed a cancer-related VTE risk assessment tool with Delphi and AHP in China. The extensive study involved consultation with experts to identify specific risk factors relevant to the Chinese population. AHP was used to determine a weighted score for each indicator to establish an effective risk threshold. Our study has successfully addressed a crucial gap in our population, where foreign tools are the only available options. In this study, we invited 23 experts in various fields, including clinical Pharmacy, Pharmaceutical Affairs, Oncology, Surgery, and Internal medicine. Among them, 20 held doctorates or master’s degrees. Our study’s tool demonstrated good reliability, with authority coefficients (*Cr*) of 0.89 and 0.90 in the two survey rounds. These coefficients indicate that the experts involved in our study had high authority. Generally, a coefficient exceeding 0.70 is considered reliable. Additionally, *Kendall’s* concordance coefficient *W* was 0.190 and 0.132 in the two-round survey, respectively, indicating that all experts had a consistent and high opinion [21].

A few strengths of our research are worth noting. First and foremost, we have created a comprehensive scale for evaluating the danger of cancer-associated VTE that factors in a broader range of risk elements, including those that can decrease the likelihood of VTE, not just those that increase it. Previous research has indicated that anticoagulants can reduce the risk of VTE [16]. Furthermore, evidence-based reviews have demonstrated that the risk of VTE in Western populations is more significant than in Asian populations. By combining the weight value of the Asian population and the use of anticoagulants, we calculated scores of 0.0046 and 0.0562 for the two factors, respectively. Consequently, our research assigned a negative score of -1 to the Asian population and − 6 to anticoagulant use, a distinct feature compared to other assessment tools. This is the only tool that uses a negative score after the “IMPEDE and SAVED score.” Our tool offers a more precise VTE risk assessment for cancer patients.

Secondly, we have modified the VTE risk score related to BMI. According to European standards, BMI > 25 kg/m^2^ is considered overweight, while that of China is > 24 kg/m^2^. Similarly, BMI > 30 kg/m^2^ is classified as obese in Europe, while in China, it is 28 kg/m^2^ or higher. Hence, BMI > 24 kg/m^2^ and ≥ 28 kg/m^2^ are used as risk factors for BMI in this study, with weights of 0.0059 and 0.0097, respectively, due to previous research indicating that higher BMIs increase VTE risk. Our study used a novel approach to assign 1 and 2 points to differentiate between the two risk factors, which has distinct advantages over other methods. The reasonable setting of the BMI threshold can more accurately evaluate patients’ VTE scores, increase the specificity of risk assessment tools, and be more suitable for the Chinese population.

Third, one of our unique contributions is the development of a new risk metric called “Plateau(Altitude ≥ 2500 m),” which is not found in other tools. It is a vital part of our thorough risk factor analysis, and we guarantee a sufficient sample size to determine VTE risk levels associated with tumors. This tool is specifically designed for clinical research purposes.

Fourth, open-ended questions were asked to gain deeper insight into the indicator during each round. This helped to define indicators and provide guidance for satisfactory practice, resulting in a more suitable index system for risk assessment tools.

However, the present study has also a few limitations. At first, Experts were only invited from 5 provinces and 12 hospitals in China, with potential experts from other regions not included. Furthermore, face-to-face discussions were not provided to address differing views. Further research is necessary before implementing this tool in practice. Clinical cases will be included to establish VTE risk thresholds and differentiate between high and low risk. Reliability and validity will be verified, and existing tools will be compared.

## Conclusion

In summary, this study centers on the characteristics of the Chinese population. We utilized the Delphi-AHP methodology to develop a VTE risk assessment instrument specifically for cancer patients in China. The tool encompasses 39 different factors. After two consultative rounds with Delphi experts, we confirmed the tool’s precision and dependability, with a significant degree of credibility and consistency.

## Data Availability

All data generated or analysed during this study are included in this published article.
